# Current status of Bayesian clinical trials for oncology, 2020

**DOI:** 10.1016/j.conctc.2020.100658

**Published:** 2020-10-01

**Authors:** Martha Fors, Paloma González

**Affiliations:** a Universidad de Las Américas. Redondel del Ciclista, Antigua Vía a Nayón, Quito EC 170124, Ecuador; b Universidad de Las Américas. Redondel del Ciclista, Antigua Vía a Nayón, Quito EC 170124, Ecuador

## Abstract

Bayesian methods had established a foothold in developing therapies in oncology trials. Methods: We identified clinical trials posted on the ClinicalTrials.gov database focused on Oncology trials with a Bayesian approach in their design. Differences in study characteristics such as design, study phase, randomization, masking, purpose of study, main outcomes, gender, age and funding involvement according to Bayesian approach were assessed using Chi-squared or Fisher's exact tests. Results: We identified 225 studies with Bayesian components in their design addressing oncological diseases. The most common designs were Bayesian Toxicity Monitoring (26.4%), Model-based designs (36%) Model-assisted designs (8%). Statistical methods such as Bayesian logistic regression model (59.4%), Bayesian piecewise exponential survival regression (10.9%) and the Continual reassessment method (9.4%) were the most used. Conclusions: Bayesian trials are more common in the early phases of drug development specifically in phase II trials (43.6%). Cancer institutes or Hospitals funded most of the studies retrieved. This type of design has increased over time and represent an innovative means of increasing trial efficiency.

## 1 Introduction

Randomized controlled trials (RCTs) have long been recognized as the gold standard for the evaluation of the efficacy and safety of clinical interventions, valued for their statistical rigor and methods to avoid bias. Frequentist statistical framework has dominated the field of clinical trials over the past six decades but nowadays, Bayesian designs have become increasingly used in clinical trials, particularly in the early phase of development clinical trials which are clinical investigations that examine and evaluate safety and efficacy of a new drug used in human subjects [[1], [2], [3]].

Bayesian inference is conditioned on the data and not on the design, so it can still maintain validity as long as the prior distribution and the probability model are correctly specified. It appropriates different levels of variability naturally under the hierarchical model assumption and allows for the incorporation of information of two types: that which accumulated in the trial and that which was obtained outside of the trial. Incorporating both types of information into the analysis strengthens the evidence for making an inference [4].

The continuous learning that is possible in the Bayesian approach enables investigators to modify trials in midcourse. Modifications include stopping the trial, adding and dropping treatment arms, and extending accrual beyond that originally targeted when the answer to the question posed is not satisfactorily known [5].

There is an interest in the Bayesian approach, highlighted by the FDA's commitment to facilitate the advancement and use of complex adaptive, Bayesian, and other novel clinical trial designs. In 2004, the FDA issued a Critical Path Initiative report stating that “The medical product development process is no longer able to keep pace with basic scientific innovation. Only a concerted effort to apply the new biomedical science to medical product development will succeed in modernizing the critical path [6].

The way Oncology clinical trials are designed and performed has changed over time. cancer therapeutic research has largely shifted from a focus on cytotoxic agents to newer drugs that act through inhibiting cancer cell growth and survival mechanisms while protecting healthy cells to the extent possible [7].

Evaluation of new therapies for cancer has suffered a paradigm shift in the last years. The use of innovative and more efficient designs is a priority for the scientific community; nevertheless, the use of this kind of design is not yet widespread [8].Traditional clinical trials require specification of the sample size in advance. This can be inefficient when limited information is available at the design stage, especially regarding the likely effect size. Bayesian approach has been used to answer more treatment questions and to foster more efficiently and novel designs and their performance in less time [9]. In general, adaptive clinical trial designs are easier to implement within the Bayesian framework.

ClinicalTrials.gov is a Web-based resource that provides patients, their family members, health care professionals, researchers, and the public with easy access to information on publicly and privately supported clinical studies on a wide range of diseases and conditions. The Web site is maintained by the National Library of Medicine (NLM) at the National Institutes of Health (NIH) [10].

ClinicalTrials.gov is designed to provide a public listing of initiated, ongoing, and completed studies, and to serve as a source of summary results information to complement the medical literature. The original focus was on facilitating identification and retrieval of information about specific studies on investigational drug products for potential study participants [11]. It is considered the world's largest clinical trial registry, public and accessible to all citizens [12].

In this cross-sectional study, we aimed to characterize the main characteristics of Bayesian Oncology Clinical trials in ClinicalTrials.gov for a 20-year period (1990–2020) through a systematic analysis of registered trials.

## 2 Methods

### 2.1 Study design

This is a cross-sectional analysis, including all interventional studies that were registered on clinicaltrials.gov in 2020 with a Bayesian approach in the design or analysis.

### 2.2 Procedures

We queried ClinicalTrials.gov for the terms “Oncology, Cancer, Tumors” in the title and condition or disease. In other terms we included “Bayesian” and search manually each trial looking for information about Bayesian approach employed. Using this search strategy, 225 trials were identified. The search was restricted to interventional trials. Two reviewers (MF and PG) extracted data and checked each other's work for accuracy.

### 2.3 Inclusion criteria

1 Trial documentation available
2 .Clinical trials of any phase
3 .Trial investigating an intervention(s) on humans.
4 .Registered or published until the moment of the search.
5 .A Bayesian design clinical trial defined to be a trial with an approach for learning from evidence as it accumulates.

### 2.4 Exclusion criteria

1 Observational studies

Definitions for variables collected in the ClinicalTrials.gov database are available at http://prsinfo.clinicaltrials.gov/definitions.html. The extracted data elements included the following:

## 3 Classification of Bayesian approaches

### 3.1 Dose finding

•**Model-based designs** included the following methods Bayesian Continual reassessment method (B -CRM), Modified continual reassessment method (mCRM), Bayesian logistic regression model (BLRM), Bayesian Time-to-event continual reassessment method (TITE-CRM), Bayesian model averaging continual reassessment method (BMA-CRM).

•**Model-assisted designs** included Bayesian optimal interval (BOIN).

### 3.2 Efficacy

• **Model-based designs** included Bayesian piecewise exponential survival regression.

### 3.3 Pharmacokinetics parameters

•Maximum A Posteriori Bayesian Estimation. (MAP).

### 3.4 Bayesian Toxicity Monitoring

• **Sequential monitoring** as the Bayesian method of Thall, Simon and Estey [13], Bayesian method of Thall and Sung [14] and Bayesian method of Thall and Cook [15] and comprised Bayesian stopping boundaries and other monitoring approaches.

**No specified Bayesian approach** included those studies that did not specify what kind of Bayesian methods were employed. The most frequent terms found were “Bayesian model”, “Bayesian approach” and “Bayesian method”.

### 3.5 Classification of funding involvement

For analysis purposes of this research, we reclassified the information of selected trials in three groups: Industry, Academy and National institutes of Cancer or Hospitals. Funding source was defined as industry if the lead sponsor was from industry, as academy if the lead sponsor was from a university and as National institutes of Cancer or Hospitals if the lead sponsor was from one of these institutions.

### 3.6 Phases

Trials were classified as early phase (phase 0, I, or I/II), late phase (phase II/III, III, or IV).

### 3.7 Intervention model

Types of intervention models include single group assignment, parallel assignment, cross-over assignment, and sequential assignment.

### 3.8 Allocation

The types of allocation are randomized allocation, nonrandomized and not applicable in case of one group of treatment.

### 3.9 Masking

Types of masking include open label, single blind masking, double-blind masking and triple-blind masking.

### 3.10 Purpose

Treatment, Supportive care, Diagnostic, Prevention and Other.

### 3.11 Main outcomes

**Efficacy** comprised Complete response, Overall survival, Progression-free survival Relapse-free survival and Time to Disease Progression.

**Toxicity** included Dose limited toxicity and incidence of adverse events.

**Dose finding** included those studies were Maximum Tolerated Dose was assessed.

### 3.12 Recruitment status

Not yet recruiting: The study has not started recruiting participants.

Recruiting: The study is currently recruiting participants.

Active, not recruiting: The study is ongoing, and participants are receiving an intervention or being examined, but potential participants are not currently being recruited or enrolled.

Terminated: The study has stopped early and will not start again.

Completed: The study has ended normally, and participants are no longer being examined or treated.

Withdrawn: The study stopped early, before enrolling its first participant.

Unknown: A study on ClinicalTrials.gov whose last known status was recruiting; not yet recruiting; or active, not recruiting but that has passed its completion date, and the status has not been last verified within the past 2 years.

### 3.13 Intervention

Interventions include drugs, medical devices, procedures, vaccines, and other products that are either investigational or already available.

### 3.14 Age or age group

The age groups were child (birth-17), adults and older adults (more than 18 years old) and all ages.

### 3.15 Gender

A type of eligibility criteria that indicates whether eligibility to participate in a clinical study is based a person's self-representation of gender identity or gender.

### 3.16 Enrollment/sample size

The number of participants in a clinical study. The “estimated” enrollment is the target number of participants that the researchers need for the study.

### 3.17 Statistical analysis

All elements were extracted directly from the database, which contains raw, row-by-row data for all registry records into a comma-separated values (csv) data file. We performed a descriptive analysis of clinical trials registered between 1990 and 2020 in the ClinicalTrials.gov database.

Descriptive statistics were primarily used to summarize the trial characteristics: categorical variables are reported as frequencies and percentages, while continuous variables are reported as mean and standard deviation. The Fisher's exact test was used to compare trial characteristics. All statistical tests were two-sided with a statistical significance at the 0.05 level. All the data were analyzed using SPSS 24.0.

## 4 Results

**Fig. 1:**
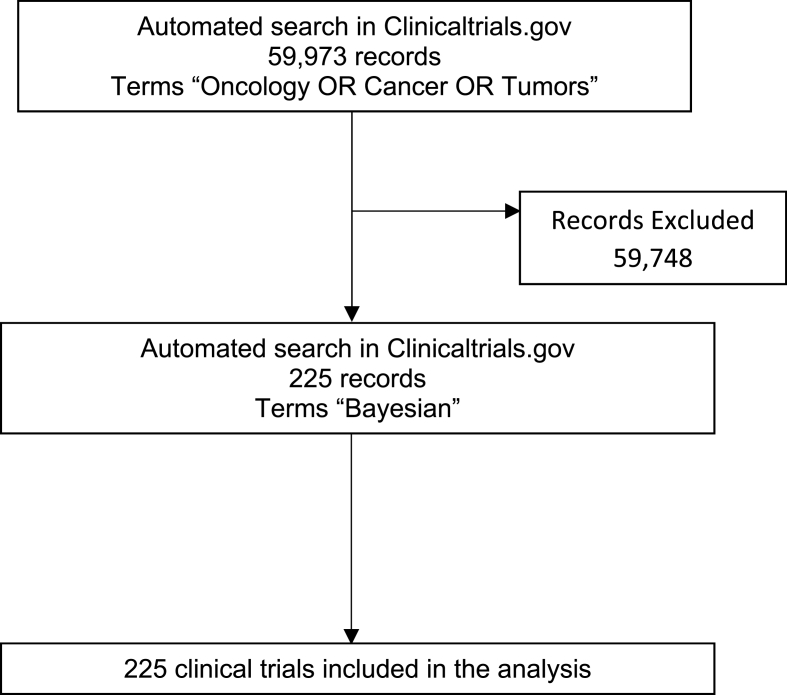
Flowchart of the review.

From the total 329,502 studies registered in the database, 59,973 were Oncology trials and from them 225 interventional studies (0.4%) were eligible for inclusion in our analyses (Bayesian approach). The trial selection process is shown in Fig. 1.

Most of the studies were in an early phase development (95.1%). Single group assignment design was the most frequent with 65.3% meaning that all participants received the same intervention and the majority of them were Open labeled (97.8%). Randomization was not applicable in 130 out of 225 studies (57.8%). 96.4% of Oncology trials evaluated disease treatment versus 1.3% supportive care. Main outcome related to efficacy (48.4%) was found in almost half of the studies while looking for the Dose Limiting Toxicity or adverse events (Toxicity) reached 31.6%. Among the 225 eligible clinical trials, 160 (71.1%) trials were evaluating Drugs, followed by studies evaluating Biological products (13.3%). We found that phase, interventional model, allocation and main outcome are significant associated with the Bayesian designed used. (Chi-Squared test, p-value < 0.05). Bayesian designs are more likely to be used in early phases than in late phases and are less likely to be used in parallel or crossover clinical trials (Table 1).

**Table 1:** Main characteristics of clinical trials.

	Credible interval (BCI)	Toxicity monitoring	Model-based designs	Model-assisted designs	No specified Bayesian approach	Total	p value
	No. (%)	No. (%)	No. (%)	No. (%)	No. (%)	No. (%)	
**Phase**							
Early Phase	16 (100.0)	57 (96.6)	63 (98.4)	18 (100.0)	60 (98.2.0)	214 (95.1)	0.05
Late phase	0 (0.0)	0 (0.0)	0 (0.0)	0 (0.0)	5 (7.4)	5 (2.2)	
Not Applicable	0 (0.0)	2 (3.4)	1 (1.6)	0 (0.0)	3 (4.4)	6 (2.7)	
**Interventional model**							
Single group assignment	8 (50.0)	48 (81.4)	39 (60.9)	13 (72.2)	39 (57.4)	**147(65.3)**	0.00
Paralell	8 (50.0)	11 (18.6)	18 (28.1)	1 (5.6)	26 (38.2)	64 (28.4)	
Sequential assignment	0 (0.0)	0 (0.0)	7 (10.9)	4 (22,2)	2 (2.9)	13 (5.8)	
Crossover	0 (0.0)	0 (0.0)	0 (0.0)	0 (0.0)	1 (1.5)	1 (0.4)	
**Masking**							
Open label	16 (100.0)	59 (100.0)	62 (96.9)	18 (100.0)	65 (95.6)	**220(97.8)**	0.44
Single blinded	0 (0.0)	0 (0.0)	1 (1.6)	0 (0.0)	0 (0.0)	1 (0.4)	
Double blinded	0 (0.0)	0 (0.0)	0 (0.0)	0 (0.0)	3 (4.4)	3 (1.3)	
Triple blinded	0 (0.0)	0 (0.0)	1 (1.6)	0 (0.0)	0 (0.0)	1 (0.4)	
**Allocation**							
Non-randomized	4 (25.0)	9 (15.3)	26 (40.6)	4 (22.2)	13 (9.1)	56 (24.9)	0.00
Randomized	4 (25.0)	4 (6.8)	8 (12.5)	1 (5.6)	22 (32.4)	39 (17.3)	
Not applicable	8 (50,0)	46 (78.0)	30 (46.9)	13 (72.2)	33 (48.5)	**130(57.8)**	
**Purpose**							
Treatment	15 (93.8)	58 (98.3)	62 (96.9)	17 (94.4)	65 (95.6)	**217(96.4)**	0.66
Supportive care	0 (0.0)	1 (1.7)	1 (1.6)	1 (5.6)	0 (0.0)	3 (1.3)	
Diagnostic	1 (6.3)	0 (0.0)	0 (0.0)	0 (0.0)	1 (1.5)	2 (0.9)	
Prevention	0 (0.0)	0 (0.0)	0 (0.0)	0 (0.0)	1 (1.5)	1 (0.4)	
Other	0 (0.0)	0 (0.0)	1 (1.6)	0 (0.0)	1 (1.5)	2 (0.9)	
**Main outcome**							
Efficacy	16 (100.0)	39 (66.1)	14 (21.9)	0 (0.0)	40 (58.8)	**109(48.4)**	0.00
Toxicity	00 (0.0)	12 (20.3)	37 (57.8)	6 (33.3)	16 (23.5)	**71(31.6)**	
Dose finding	0 (0.0)	8 (13.6)	13 (20.3)	12 (66.7)	12 (17.6)	45 (20.0)	
**Intervention**							
Drug	10 (62.5)	41 (69.5)	44 (68.8)	12 (66.7)	53 (77.9)	**160(71.1)**	0.09
Biological	1 (6.3)	9 (15.3)	13 (20.3)	2 (11.1)	5 (7.4)	**30(13.3)**	
Procedure	2 (12.5)	1 (1.7)	5 (7.8)	3 (16.7)	4 (5.9)	15 (6.7)	
Radiation	0 (0.0)	3 (5.1)	1 (1,6)	0 (0.0)	4 (5.9)	8 (3.6)	
Behavioral	0 (0.0)	0 (0.0)	0 (0.0)	0 (0.0)	1 (1.5)	1 (0.4)	
Device	0 (0.0)	0 (0.0)	0 (0.0)	0 (0.0)	1 (1.5)	1 (0.4)	
Other	3 (18.8)	5 (8.5)	1 (1.6)	1 (5.6)	0 (0.0)	10 (4.4)	
**Total**	**16 (7.1)**	**59(26.2)**	**64(28.4)**	**18(8.0)**	**68(30.2)**	**225 (100.0)**	

**Fig. 2:**
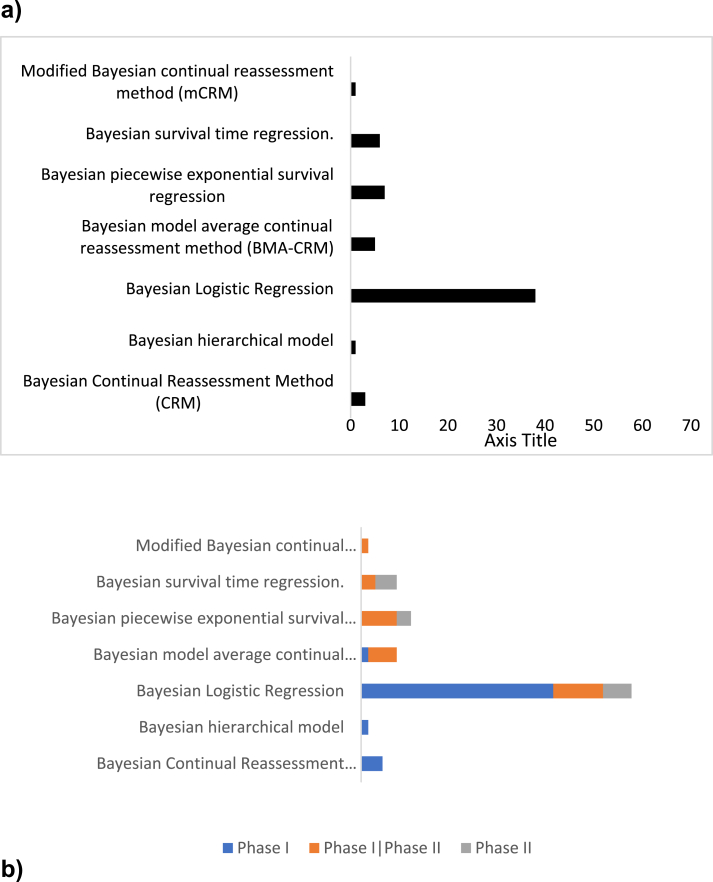
Model based Bayesian methods a) Number and Percentage of Model based designs. b) Model based designs according Phase of development.

Model-based designs were accounted for 28.4% (64/225). From them the most frequent methods were the Bayesian Logistic regression with 59.4% and the Bayesian piecewise exponential survival regression with 10.9%. Model average continual reassessment method and Bayesian piecewise exponential survival regression were the most common Bayesian methods implemented in phases I/II (Fig. 2).

Approximately one-quarter of all trials were using Bayesian Toxicity Monitoring (26.2%) approach, the most frequent was the Bayesian method of Thall, Simon, and Estey. Among the Model-assisted methods, Bayesian optimal interval (BOIN) was the most frequent design (94.4%). Around 30% of the studies has not declared a specific Bayesian method.

144 studies (64.0%) reported National Institutes of Cancer or Hospitals as the main source of funding involvement while 60 (26.7%) reported industry involvement (Table 2).

**Table 2:** Type of funding.

Type of funding	No.	%
National Institutes of Cancer/Hospitals	144	64.0
Industry	60	26.7
Academy	21	9.3
Total	225	100.0

**Fig. 3:**
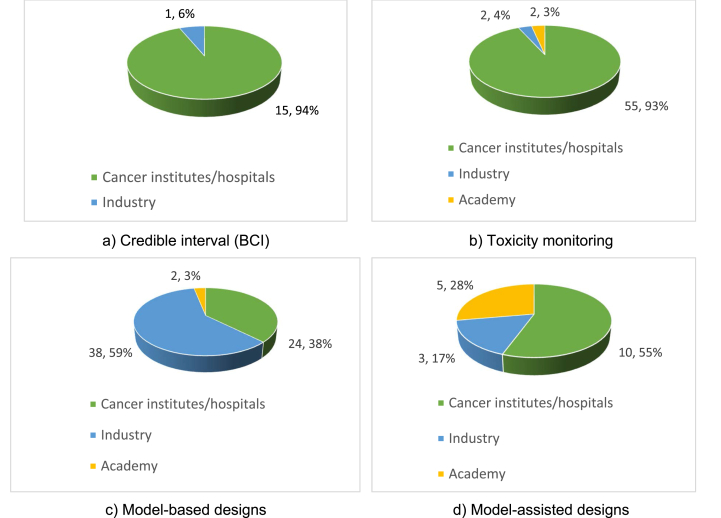
Type of Bayesian designs according type of centers funding the studies.

Fig. 3 shows Bayesian design segregated by trial funder. After the reclassification of source of funding, we found that Cancer institutes are using this kind of design more than other funding institutions.

Most Bayesian studies included both male and female participants (84.4%). 103 (45.8%) studies were in the process of recruiting and only 5.3% studies were already completed. Among these, 66.6% of them had reported results of trial on ClinicalTrials.gov.

Average years of clinical trial duration was of 4.63 with SD 3.19. Mean of participants per trial was 86 CI95% (71.1–101.3).

**Fig. 4:**
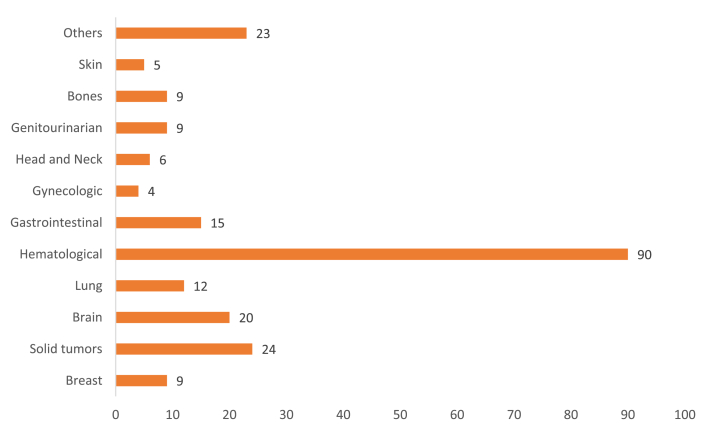
Localization of cancer.

The therapeutic area of all the intervention was manually sorted and the distribution of therapeutic area is presented in Fig. 4. The top 5 therapeutic areas were Hematological cancers (15.9%), Solid tumors (10.6%), Brain tumors (12.7%), Gastrointestinal (10.1%), and Lung cancer (8.6%).

## 5 Discussion

This review explores the use of Bayesian designs in published and publicly available Oncology trials found in Clinicaltrials.gov. registry as the only information source. We demonstrated that Bayesian trials were predominantly used in early-phase studies with a generally small proportion of randomized and parallel assignment studies. Most of them were one group assignment and with open label design. Consequently, these studies were dominant (66.8%) in our study.

Published literature like our review is limited for comparing obtained results with those of other authors, but there is a research performed at the MD Anderson Cancer Center that has studied Bayesian approach in their clinical trials in 2019. They reported that Bayesian trials were more common in phase I/II trials [16].

There are some reviews regarding Oncology trials but not specifically with a Bayesian approach in the design or analysis that have reported similar results about the main characteristics of clinical trials in this medical specialty [17,18]. It is common in Oncology trials to find this kind of phases frequently [19].

We found that approximately half of the trials studied efficacy (response to treatment) meanwhile other authors reported more studies monitoring toxicity [13].

Compared with trials for supportive care, prevention or diagnostic, more trials were treatment-oriented, mainly focused on new drugs evaluation.

Our results have shown that the Bayesian logistic regression model was the most used in phases I although it can be found in other early phases. The BLRM is another modification of the CRM which updates the estimate of the dose–toxicity curve based on the accumulating data and assigns the next cohort of patients to the currently estimated “optimal” dose [20].

Model average continual reassessment method and Bayesian piecewise exponential survival regression were the most common Bayesian methods implemented in phases I/II. In general, CRM is a model-based design for phase I trials, which aims to find the maximum tolerated dose (MTD) of a new therapy. The CRM has been shown to be more accurate in targeting the MTD than traditional rule-based approaches such as the 3 + 3 design, which is used in most phase I trials [21].

Bayesian method of Thall, Simon, and Estey is Bayesian sequential monitoring designs for single-arm clinical trials. These authors presented a Bayesian decision criteria and monitoring boundaries for early termination of studies with unacceptably high rates of adverse outcomes or with low rates of desirable outcomes [13].

Bayesian Optimal Interval (BOIN) was the most frequent design among assisted-models designs. One advantage of interval designs is that they are simple to implement in practice. Because the interval is prespecified, during trial conduct, the decision of which dose to administer to the next cohort of patients does not require complicated computations, but only a simple comparison of the observed toxicity rate at the current dose with the prespecified interval boundaries [22].

It appears that in the academic community the interest, or the knowledge, for using Bayesian designs are limited. Of the 225 trials only a minority (9.8%) evaluated new therapeutic alternatives funded by universities. It seems that researchers of Institutes of cancer are leading the way with innovative Bayesian approaches in the treatment in cancer related diseases.

According to the study performed by Califf et al. reported that a great number of studies are funded by organizations other than industry or the National Institutes of Health. Most trials registered were relatively small samples, with the average number of 80 participants per trial. Califf also reported that most interventional trials registered between 2007 and 2010 were small, with 62% enrolling 100 or fewer participants [23].

More than half trials were completed, and 66.6% of trials had results available on the ClinicalTrials.gov, which in our consideration is a step forward clinical trials transparency.

Several clinical trials are now running with design of Bayesian approach, maybe the reason is the computational advantage and complex issue in data analysis give the up-gradation of Bayesian approach over classical methods [24].

## 6 Conclusions

Bayesian trials are more common in the early phases of drug development and represent an innovative means of increasing trial efficiency. This type of design has increased over time among researchers working at cancer institutes or hospitals. Optimization of clinical trials is one of possible approaches to speed the drug development process making better use of all available information. Bayesian statistics provides the opportunity to make clinical trials more efficient.

### Strengths

We have provided a comprehensive descriptive and analytic assessment of the current information regarding Oncology Bayesian clinical trials in the ClinicalTrials.gov registry from 1990 to February 2020. We followed a strict analysis to arrive at reliable results.

### Limitations

There are some limitations to this analysis. Not all the information regarding design was clear or complete, some studies had limited specification on the kind of Bayesian approach was used. Not all studies had a regularly updated information, which may lead to inaccurately reported data. There are some clinical trials worldwide that are not registered in this database implying that the clinical trials included in this manuscript may not be a representative sample of studies with this design.

### Data availability

The raw data that support the findings of this study are available from the corresponding author upon reasonable request.

## Author contributions

M.F., P.G. conceived the research, M.F. processed data; M.F., P.G analyzed the data, wrote and contributed to manuscript editing.

## Declaration of competing interest

The authors declare no competing interests.
